# AAA Research Methodology I: Overcoming Linguistic and Cultural Barriers in Aging Research Among Asians: Presenter Discussion

**DOI:** 10.1093/geroni/igaa057.3519

**Published:** 2020-12-16

**Authors:** MacKenzie Hughes, Clara Coblenz, Emily Smith, Shevaun Neupert, Ann Pearman

**Affiliations:** 1 Georgia Institute of Technology, Atlanta, Georgia, United States; 2 North Carolina State University, Raleigh, North Carolina, United States

## Abstract

Slowing the spread of COVID-19 depends on public adherence to precautionary actions, such as wearing masks. The Health Belief Model (Rosenstock, 1974) suggests the likelihood of using precautionary measures depends on perceived susceptibility, the severity of a disease, and whether effective measures can be taken to reduce the perceived threat of a disease. This daily diary study focused on identifying predictors of daily precautionary behavior in older persons. Between April 1 and June 26, 2020, 261 adults ages 55-79 (M = 64.29, SD = 5.20) completed up to 30 consecutive days of online diaries. We examined whether perceived risk, COVID-19 knowledge, fake news beliefs, information seeking, disruption to routine, in-person interactions, and leaving the house predicted the number of daily precautions participants engaged in. Multilevel modeling was used to examine within-person fluctuations in precautions as well as change in precautions from one day to the next. People who reported higher education, scored higher on the COVID-19 knowledge quiz, had lower fake news beliefs, and perceived a higher risk of contracting COVID-19 endorsed more precautions. At the daily level, increases in the number of in-person interactions, leaving home, and disruption to daily routine were each associated with decreases in precautionary behaviors. Concurrent day and lagged models showed significant interactions between information seeking and perceived risk, suggesting increases in information seeking are related to increases in precautions for those who consider their risk to be low. Findings highlight potentially intervenable factors that influence older adults’ daily decision making related to precautionary actions.

