# Erratum to: AAA Research Methodology I: Overcoming Linguistic and Cultural Barriers in Aging Research Among Asians: Presenter Discussion

**DOI:** 10.1093/geroni/igab007

**Published:** 2021-04-22

**Authors:** MacKenzie Hughes, Clara Coblenz, Emily Smith, Shevaun Neupert, Ann Pearman

**Affiliations:** 1 Georgia Institute of Technology, Atlanta, Georgia, United States; 2 North Carolina State University, Raleigh, North Carolina, United States

In the published abstract by Hughes, Coblez, Smith, Neupert, and Peraman, doi: 10.1093/geroni/igaa057.3519, the abstract title was erroneously listed as “AAA Research Methodology I: Overcoming Linguistic and Cultural Barriers in Aging Research Among Asians: Presenter Discussion”. The correct title should be: “Predictors of Precautionary Actions by Older Adults During COVID-19: A Daily Diary Study”. This has since been corrected.

